# Assessing the measurement invariance of the 10-item Centre for Epidemiological Studies Depression Scale and Beck Anxiety Inventory questionnaires across people living with HIV/AIDS and healthy people

**DOI:** 10.1186/s40359-021-00546-1

**Published:** 2021-03-09

**Authors:** Zahra Bagheri, Pegah Noorshargh, Zahra Shahsavar, Peyman Jafari

**Affiliations:** 1 Department of Biostatistics, School of Medicine, Shiraz University of Medical Sciences, Shiraz, Iran; 2 Department of English Language, Shiraz University of Medical Sciences, Shiraz, Iran

**Keywords:** Depression, Anxiety, Measurement invariance, PLWHA, Healthy population

## Abstract

**Background:**

Recently, extensive research has been reported the higher rate of depression and anxiety among people living with HIV/AIDS (PLWHAs) as compared to the general population. However, no single study has been carried out to investigate whether this disparity is a real difference or it happens due to lack of measurement invariance. This study aims to assess the measurement invariance of the Beck Anxiety Inventory (BAI) and 10-item Centre for Epidemiological Studies Depression Scale (CESD-10) questionnaires across PLWHAs and healthy individuals.

**Methods:**

One hundred and fifty PLWHAs and 500 healthy individuals filled out the Persian version of the BAI and CESD-10 questionnaires. Multi-group multiple-indicators multiple-causes model (MG-MIMIC) was used to assess measurement invariance across PLWHAs and healthy people.

**Results:**

Our findings revealed that PLWHAs and healthy individuals perceived the meaning of all the items in the BAI and CESD-10 questionnaires similarly. In addition, although depression scores were significantly higher in PLWHAs as opposed to the healthy individuals, no significant difference was observed in anxiety scores of these two groups.

**Conclusions:**

The current study suggests that the BAI and CESD-10 are invariant measures across PLWHAs and healthy people which can be used for meaningful cross-group comparison. Therefore, in comparison to healthy individuals, higher depression score of PLWHAs is a real difference. It is highly recommended that health professionals develop therapeutic interventions and psychological supports to promote the mental health of PLWHAs which alleviate their depressive symptoms.

## Background

A growing body of literature has highlighted that people living with HIV/AIDS (PLWHAs) experience a wide variety of distressing events such as complicated therapeutic regimes, disease exacerbation, shortened life expectancy, presence of pain, poor social and family support, financial burden as well as fear of disclosure and stigma [1–8]. Accordingly, it is not surprising that depression and anxiety are highly prevalent among PLWHAs [2, 9–17]. The diagnosis and treatment of depression and anxiety in PLWHAs has received special attention in the past decade. The importance of this issue is underlined by the fact that the underdiagnosis and undertreatment of these psychological disorders usher in lower quality of life, poor adherence to HIV medications, faster disease progression, deterioration in immunological function, suicidal ideation, greater sexual risk behaviors, and marital conflict [10, 11, 13, 14, 18–26]. In general, the prevalence rate of depression and anxiety has been estimated from 3.2 to 45% and from 1.27 to 53% among PLWHAs, respectively [2, 9, 12, 27, 28]. This diversity in prevalence rates can be contributed to various populations of patients with HIV/AIDS in different studies, research settings and more importantly the methods and criteria used for the assessment and diagnosis of depression and anxiety.

Using Self-administered questionnaires is the most common method to measure depression and anxiety in the clinical and research settings. In recent years, a number of questionnaires have been introduced to assess these aforementioned psychological disorders in PLWHAs [29–33]. In addition to depression and anxiety diagnosis, researchers often use such measures to compare mean level of depression and anxiety between PLWHAs and other subpopulations, especially healthy population. As shown in previous studies, compared to the healthy population, PLWHAs had significantly higher depression and anxiety scores [1–3, 6]. However, the validity of such cross-group comparisons depends on an important assumption which is known as measurement invariance. Measurement invariance means that different respondents from different groups perceive the meaning of the items in a given questionnaire similarly [34]. When measurement invariance does not hold, it is not clear whether the observed disparity in depression and anxiety scores between PLWHAs and healthy population is a real difference in the underlying construct of interest, or it is due to an artificial effect of different interpretations of items by PLWHAs and healthy individuals. Furthermore, pioneer researchers pointed out that in some subpopulations of chronic patients including HIV/AIDS, disentangling symptoms such as fatigue, sleep difficulties, and pain that are attributable to depression and anxiety as opposed to those that are owing to the disease or medication side effects is a challenging issue and segregation of their origin is onerous [13, 35]. Hence, it is of critical importance to investigate the measurement invariance of depression/anxiety questionnaires in PLWHA samples. If PLWHAs and healthy population perceive the meaning of the items of studied questionnaires in the same way, it can be concluded that the symptoms of depression and anxiety are not misinterpreted as the symptoms of the disease or medication side effects [35].

Although some studies have been carried out to investigate the measurement invariance of depression/anxiety questionnaires across various patient groups such as breast cancer, migration, neurologic patients, and healthy population [35–37], to the best of our knowledge, no study has been conducted to examine the measurement invariance of these questionnaires across PLWHAs and healthy population. To fill this gap, this study aims to evaluate the measurement invariance of the Beck Anxiety Inventory (BAI) and 10-item Centre for Epidemiological Studies Depression Scale (CESD-10) instruments across PLWHAs and healthy population.

## Methods

### Participants

All PLWHAs who referred to a voluntary counselling and testing (VCT) centre for preventive and medical care services, were invited to the study between February and June 2015 in Shiraz (Southern Iran). During the study period, 150 individuals participated in the study after filling out the consent forms. Patients who could read and write were included in the study; while those with neurocognitive impairment (2.4%) were excluded. We collected some socio-demographic characteristics such as age, gender, job status, and education. In order to assess depression and anxiety of the patients, the Persian version of the CESD-10 and the BAI questionnaires were used, respectively.

Furthermore, a random sample of 500 healthy individuals were selected based on a two-stage cluster sampling technique across four educational districts located in diverse socioeconomic areas in the city of Shiraz. The data was collected from September to December 2015. In the first stage, a random sample of schools was selected from each educational district. Then, we selected one or two classes out of each school. A trained researcher distributed the CESD-10, BAI instruments, and the informed consent form to all students and asked them to give the questionnaires to their parents. Approximately, 70% (500 out of 700) of parents filled out the questionnaires at home after signing the informed consent form. In a couple of days, the completed questionnaires were turned back to school by children.

### Instruments

#### Beck Anxiety Inventory (BAI)

The Persian version of the BAI questionnaire was used to measure anxiety in both PLWHAs and healthy individuals. The BAI is a self-report questionnaire that can reliably discriminate anxiety from depression. It comprises 21 anxiety symptoms that affected the participants during their last week. Individuals responded to the items on a 4-point Likert scale (from 0 = 'not at all' to 3 = 'severely, I could barely stand it'). The total score is the sum of all the items and ranges from 0 to 63, with a higher score showing greater anxiety [38]. The instrument was translated and validated previously in Persian which showed excellent internal consistency (Cronbach’s alpha = 0.92) and acceptable test–retest reliability (r = 0.72). The Persian version of the BAI also had stronger correlation (r = 0.40–0.44) with measures of anxiety as compared to measures of depression (r = 0.21) [39, 40]. The main reasons for selecting the BAI are its simplicity, briefness and widely use in clinical research. The original (English) version of the questionnaire is available at the following link: https://www.sciencedirect.com/topics/medicine-and-dentistry/beck-anxiety-inventory.

#### 10-item Centre for Epidemiological Studies-Depression Scale (CESD-10)

To measure the depression status of PLWHAs and healthy individuals, the Persian version of the CESD-10 was applied [41]. It has been shown that the Persian version of the CESD-10 had acceptable internal consistency (Cronbach’s alpha = 0.85) and test–retest reliability (r = 0.65). It also showed good construct validity with the factor loadings of greater than 0.40 for all the items (41). According to previous research, this questionnaire is suitable to assess depression among PLWHAs. It is a short, easy to read and easy to score instrument which can reduce interview burden on the patients [33]. Moreover, it has been widely used for assessing depression symptoms in the general population. The items are scored on a 4-point Likert scale from 0 (not at all) to 3 (a lot). The total score is the sum of all the items with the possible range of 0 to 30. The higher is the total score, the greater is the degree of depressive symptoms. The original (English) version of the questionnaire is available in the article by Zhang et al. (2012) [33].

### Statistical analysis

Chi-square statistics and two-sided independent sample t test were applied to investigate whether PLWHAs and healthy people differed significantly in terms of categorical and continuous demographic characteristics, respectively. P value < 0.05 was considered as a significance level.

In the present study, the internal consistency of the CESD-10 and BAI questionnaires was assessed by Cronbach’s alpha coefficient. A coefficient equal to or greater than 0.70 was considered to be a satisfactory level of reliability. Furthermore, prior to assessing measurement invariance, categorical confirmatory factor analysis (CCFA) was used to investigate the construct validity of the CESD-10 and BAI in both groups of healthy people and PLWHAs, separately. According to Thompson, the value of 0.3 was used as a factor loading criterion in this study [42]. Since a two-factor model was originally reported for the BAI by Beck [43], both one- and two-factor models were estimated to determine the best-fitting factor model in our sample.

The measurement invariance of a questionnaire is evaluated by differential item functioning (DIF) analysis. DIF occurs when people from different groups respond differently to a particular item given the same level of latent trait of interest. Two types of DIF can be identified, namely uniform and non-uniform DIF [34]. Uniform DIF means that on the entire continuum of the latent trait, item response probabilities are higher (lower) in one group compared to the other one. In contrast, in non-uniform DIF, the direction of DIF is different in different levels of latent trait [34].

In the present study, the multi-group multiple-indicators multiple-causes model (MG-MIMIC) was used to assess the measurement invariance of the BAI and CESD-10 instruments across PLWHAs and healthy individuals. Technically, the MG-MIMIC model is an integration of multi-group confirmatory factor analysis (MGCFA) and MIMIC models that implements the same model constraints that are implicit in item response theory models [44, 45]. This means that this model imitates the concepts of uniform and non-uniform DIF in item response theory to measurement non-invariance in factor analysis approach. In this model, uniform DIF is detected when discrepancy is observed in the thresholds of a given item across the groups and non-uniform DIF is identified when the factor loading of an item differs between the groups. In addition, in this technique the effects of confounding variables can also be controlled while assessing DIF [44, 45]. In this study, the effects of age, gender and education which differed significantly between PLWHAs and healthy individuals were taken into account while examining DIF. In the MG-MIMIC model, DIF detection process is iterative and consists of serial tests of nested models, initiating with the most constrained model, consecutively relaxing cross-group equality constraints on the parameters, and then finishing up with the least constrained model.

In the first step, a baseline model which is the most constraint model is estimated. This model is fully invariant in factor loadings, thresholds, residual variance, latent trait variance and scaling factor across the groups. In this model, the only parameter which is freely estimated for both groups is the mean for latent trait, which allows estimating the group differences in underlying depression/anxiety. In step 2, the value of modification indices associated with factor loadings and thresholds in the baseline model is examined and the modification which would lead to the larger improvement in model fit is determined. If the modification index associated with thresholds of an item is larger than the others, this item is a candidate for uniform DIF. If the modification index associated with factor loading of an item is larger than the others, this may be an indication of non-uniform DIF item.

In step 3, the DIFFTEST procedure is used [46] to fit a model that relaxes the equality constraint on factor loadings across the groups relative to the baseline model. In step 4, the DIFFTEST procedure is used to fit a model that relaxes the equality constraint on item thresholds relative to the baseline model. In step 5, the chi-square values from DIFFTEST procedure for these two modifications are compared and the larger one is identified; if it is significant, that modification is accepted and the other is rejected. If relaxing of factor loading parameter leads to larger improvement of the model and it is significant, non-uniform DIF is detected. While, if relaxing of thresholds parameter results in larger improvement and it is significant, uniform DIF is detected. In step 6, the resultant model in step 5 is considered as a new baseline model with the values of modification indices being examined again and all the steps mentioned above are repeated until no significant model modification is identified.

In order to assess the goodness of fit of the CCFA and MG-MIMIC models several indices were used including Chi-square statistics, root mean square error of approximation (RMSEA), Tucker–Lewis index (TLI), and comparative fit index (CFI). Although a non-significant value of Chi-square shows acceptable model fit, this index detects even trivial differences under large sample size. Hence, the other above-mentioned fit indices should also be considered for testing goodness of fit of the model. Values of CFI and TLI > 0.90, and RMSEA < 0.08 support that the model fit well [47]. In addition, for testing the relative fit of two nested models, Δχ^2^, ΔCFI, and ΔRMSEA were used. According to Chen (2007), significant Δχ^2^, ΔCFI > − 0.01, and ΔRMSEA > 0.015 would indicate measurement non-invariance [48]. In the present study, the mean and variance-adjusted weighted least square (WLSMV) estimation procedure, which has been introduced for ordinal indicators, was applied to fit the CCFA and MG-MIMIC model using Mplus 6.1 software.

## Results

Table 1 shows the summary statistics for demographic characteristics of the PLWHAs and healthy individuals. The mean age of healthy people (42.67 ± 6.78) was significantly greater than that of PLWHAs (39.68 ± 7.92). The percentage of male were significantly higher among PLWHAs as opposed to healthy people (64% vs 50.6%). In addition, the number of individuals with above diploma-level education was significantly higher among healthy population as compared to the PLWHAs (91.4% vs 68.2%). However, there was no significant difference between healthy population and PLWHAs in terms of their employment status.

**Table 1:** Demographic characteristics of PLWHAs and healthy individuals

Variable	PLWHA	Healthy	
	Mean ± SD	Mean ± SD	P value^¶^
Age	39.68 ± 7.92	42.67 ± 6.78	< 0.001

	Number (%)	Number (%)	P value*
*Sex*			
Male	96 (64)	253 (50.6)	< 0.001
Female	54 (36)	247 (49.4)	
*Education*			
Under diploma	11 (7.3)	130 (26)	< 0.001
Above diploma	137 (91.4)	341 (68.2)	
Missing	2 (1.3)	29 (5.8)	
*Job*			
Employed	60 (40)	184 (36.8)	0.533
Unemployed	77 (51.3)	267 (53.4)	
Missing	13 (8.7)	49 (9.8)	

*p value based on Chi-square statistics

^¶^P value based on independent samples t test

Table 2 represents the results of CCFA for assessing the construct validity of the CED-10 and BAI questionnaires as well as the Cronbach’s alpha coefficient. As indicated, the magnitudes of goodness of fit indices supported the one-factor model for the CESD-10 in both healthy people (RMSEA = 0.08, CFI = 0.96, TLI = 0.94) and PLWHAs (RMSEA = 0.068, CFI = 0.97, TLI = 0.96). In addition, the factor loadings of all the items were significant and higher than 0.3 in both groups except for that of item 4 in PLWHAs which was 0.245 (The full results are not presented here due to the space limitation). Interestingly, for the BAI questionnaire, both one- and two-factor models fit equally well in healthy people (RMSEA = 0.057, CFI = 0.95, TLI = 0.94) and PLWHAs (RMSEA = 0.071, CFI = 0.94, TLI = 0.93). The factor loadings of all the items were significant and higher than 0.50 in both groups (The full results are not presented here due to the space limitation). In addition, the Cronbach’s alpha coefficients for the CESD-10 were 0.74 and 0.75 for healthy people and PLWHs, respectively. For the BAI, the Cronbach’s alpha value was 0.91 for both groups.

**Table 2:** Goodness of fit indices for CCFA model and Cronbach’s alpha coefficient for CESD-10 and BAI questionnaires in healthy people and PLWHAs

Questionnaire	Group	χ^2^ (df), p	CFI	TLI	RMSEA	Cronbach’s alpha
CESD-10	Healthy	162.62 (33), < 0.001	0.96	0.94	0.080	0.74
	PLWHA	54.36 (32), 0.008	0.97	0.96	0.068	0.75
One-factor BAI	Healthy	593.54 (189), < 0.001	0.95	0.94	0.057	0.91
	PLWHA	332.03 (189), < 0.001	0.94	0.93	0.071	0.91
Two-factor BAI	Healthy	590.96 (188), < 0.001	0.95	0.94	0.057	0.91
	PLWHA	329.53 (188), < 0.001	0.94	0.93	0.071	0.91

Table 3 shows the results of the MG-MIMIC model including the factor loadings and thresholds of the items in the CESD-10 questionnaire across PLWHAs and healthy people. As indicated the factor loadings and thresholds of all the items were equal between PLWHAs and healthy individuals which implies that there was no DIF item across the groups and measurement invariance of the CESD-10 was established. The values of fit indices (RMSEA = 0.069, CFI = 0.94, and TLI = 0.94) also confirmed that the most constraint model fit well to the data. It should be noted that although the values of modification indices associated with all factor loadings and thresholds were small in the most constraint model, we proceeded with the next steps to investigate that whether relaxing equality constraints can lead to further improvement of the model fit and any DIF item can be detected. The largest modification index among factor loadings was associated with item 6 (modification index = 4.61); consequently, the equality constraint of this factor loading was relaxed and the resultant model was compared with the baseline model based on DIFFTEST procedure. The values of Δχ^2^ (Δdf), ΔCFI, and ΔRMSEA were 1.66(1), − 0.001, 0.001, respectively, suggesting that no modification was needed and this item was not a candidate for non-uniform DIF. The largest modification index among thresholds was associated with item 9 (modification index = 2.59); hence, the equality constraint on this item thresholds was relaxed and the resulting model was compared with the baseline model based on DIFFTEST procedure. The values of Δχ^2^ (Δdf) = 5.91(3), ΔCFI = − 0.0001, and ΔRMSEA = 0.001 indicated that no significant improvement was observed and this item cannot be considered as uniform DIF. Since neither of the difference testing results produced a statistically significant improvement in model fit, we then stopped the algorithm.

**Table 3:** Item parameters and standard errors for the items in the CESD-10 questionnaire across PLWHAs and healthy population

	Factor loading	1st threshold	2nd threshold	3rd threshold
	b (SE)	τ_1_ (SE)	τ_2_ (SE)	τ_3_ (SE)
*q1: I was bothered by things that usually don't bother me*				
Healthy	0.870 (0.077)	− 1.111 (0.470)	0.007 (0.469)	0.888 (0.473)
PLWHA	0.870 (0.077)	− 1.111 (0.470)	0.007 (0.469)	0.888 (0.473)
*q2: I had trouble keeping my mind on what I was doing*				
Healthy	0.841 (0.073)	− 0.849 (0.494)	0.435 (0.492)	1.072 (0.508)
PLWHA	0.841 (0.073)	− 0.849 (0.494)	0.435 (0.492)	1.072 (0.508)
*q3: I felt depressed*				
Healthy	1.492 (0.124)	− 2.447 (0.678)	− 1.094 (0.668)	− 0.004 (0.668)
PLWHA	1.492 (0.124)	− 2.447 (0.678)	− 1.094 (0.668)	− 0.004 (0.668)
*q4: I felt that everything I did was an effort*				
Healthy	0.268 (0.049)	1.025 (0.334)	0.382 (0.330)	− 0.246 (0.331)
PLWHA	0.268 (0.049)	1.025 (0.334)	0.382 (0.330)	− 0.246 (0.331)
*q5: I felt hopeful about the future*				
Healthy	0.654 (0.062)	− 1.190 (0.398)	− 0.469 (0.396)	0.115 (0.399)
PLWHA	0.654 (0.062)	− 1.190 (0.398)	− 0.469 (0.396)	0.115 (0.399)
*q6: I felt fearful*				
Healthy	1.036 (0.087)	− 1.354 (0.539)	− 0.320 (0.537)	0.535 (0.539)
PLWHA	1.036 (0.087)	− 1.354 (0.539)	− 0.320 (0.537)	0.535 (0.539)
*q7: My sleep was restless*				
Healthy	1.006 (0.080)	− 0.443 (0.522)	0.610 (0.523)	1.488 (0.538)
PLWHA	1.006 (0.080)	− 0.443 (0.522)	0.610 (0.523)	1.488 (0.538)
*q8: I was happy*				
Healthy	0.824 (0.066)	− 1.887 (0.408)	− 0.741 (0.403)	0.298 (0.404)
PLWHA	0.824 (0.066)	− 1.887 (0.408)	− 0.741 (0.403)	0.298 (0.404)
*q9: I felt lonely*				
Healthy	1.092 (0.088)	− 1.521 (0.523)	− 0.435 (0.520)	0.505 (0.526)
PLWHA	1.092 (0.088)	− 1.521 (0.523)	− 0.435 (0.520)	0.505 (0.526)
*q10: I could not "get going"*				
Healthy	1.264 (0.116)	− 1.192 (0.592)	− 0.266 (0.593)	0.648 (0.600)
PLWHA	1.264 (0.116)	− 1.192 (0.592)	− 0.266 (0.593)	0.648 (0.600)

b: factor loading, τ_i_: threshold parameters, SE: standard error

Table 4 represents the estimated factor loadings and thresholds of the one-factor BAI's items resulting from the MG-MIMIC model for assessing DIF across PLWHAs and healthy individuals. As shown the values of factor loadings and thresholds of all the items were the same across the groups. This means that no DIF item was detected and the BAI is an invariant questionnaire across PLWHAs and healthy individuals. In addition, the values of fit indices supported the fit of the most constraint model (RMSEA = 0.064, CFI = 0.95, and TLI = 0.96). The values of modification indices associated with all factor loadings and thresholds were small in the most constraint model. Nevertheless, we continued to the next steps. The largest modification index among factor loadings was associated with item 17 (modification index = 10.69); consequently, the equality constraint of this factor loading was relaxed across the groups and the resultant model was compared with the baseline model based on DIFFTEST procedure. The values of Δχ^2^ (Δdf), ΔCFI, and ΔRMSEA were 3.41(1), − 0.0001, 0.0001, respectively, indicating that no significant improvement was observed and this item cannot be considered as non-uniform DIF. The largest modification index among thresholds was associated with item 13 (modification index = 1.54); hence, the model that relaxed equality constraint on this item threshold was fitted and compared with the baseline model based on DIFFTEST procedure. The values of Δχ^2^ (Δdf) = 3.14(3), ΔCFI = − 0.0001, and ΔRMSEA = 0.0001 suggested that no significant change was detected in model fit and this item cant not be considered as uniform DIF. Therefore, we stopped the algorithm since neither of the difference testing results produced a statistically significant improvement in model fit.

**Table 4:** Item parameters and standard errors for the items in the BAI questionnaire across PLWHAs and healthy general population

	Factor loading	1st threshold	2^nd^ threshold	3rd threshold
	b (SE)	τ_1_ (SE)	τ_2_ (SE)	τ_3_ (SE)
*q1: Numbness or tingling*				
Healthy	0.887 (0.072)	0.442 (0.481)	1.615 (0.479)	2.846 (0.518)
PLWHA	0.887 (0.072)	0.442 (0.481)	1.615 (0.479)	2.846 (0.518)
*q2: Feeling hot*				
Healthy	0.797 (0.061)	0.024 (0.441)	0.922 (0.443)	2.387 (0.446)
PLWHA	0.797 (0.061)	0.024 (0.441)	0.922 (0.443)	2.387 (0.446)
*q3: Wobbliness in legs*				
Healthy	1.089 (0.095)	0.277 (0.630)	1.025 (0.634)	2.222 (0.666)
PLWHA	1.089 (0.095)	0.277 (0.630)	1.025 (0.634)	2.222 (0.666)
*q4: Unable to relax*				
Healthy	1.115 (0.077)	− 0.521 (0.550)	0.818 (0.554)	2.083 (0.570)
PLWHA	1.115 (0.077)	− 0.521 (0.550)	0.818 (0.554)	2.083 (0.570)
*q5: Fear of worst happening*				
Healthy	0.891 (0.060)	− 0.519 (0.473)	0.459 (0.476)	1.822 (0.490)
PLWHA	0.891 (0.060)	− 0.519 (0.473)	0.459 (0.476)	1.822 (0.490)
*q6: Dizzy or lightheaded*				
Healthy	1.163 (0.086)	− 0.927 (0.671)	0.126 (0.678)	1.588 (0.731)
PLWHA	1.163 (0.086)	− 0.927 (0.671)	0.126 (0.678)	1.588 (0.731)
*q7: Heart pounding/racing*				
Healthy	1.071 (0.075)	0.430 (0.583)	1.550 (0.587)	2.716 (0.598)
PLWHA	1.071 (0.075)	0.430 (0.583)	1.550 (0.587)	2.716 (0.598)
*q8: Unsteady*				
Healthy	1.130 (0.091)	0.642 (0.587)	1.888 (0.579)	2.957 (0.629)
PLWHA	1.130 (0.091)	0.642 (0.587)	1.888 (0.579)	2.957 (0.629)
*q9: Terrified or afraid*				
Healthy	1.105 (0.087)	0.463 (0.628)	1.410 (0.619)	2.869 (0.706)
PLWHA	1.105 (0.087)	0.463 (0.628)	1.410 (0.619)	2.869 (0.706)
*q10: Nervous*				
Healthy	0.914 (0.060)	− 1.039 (0.482)	0.064 (0.481)	1.698 (0.496)
PLWHA	0.914 (0.060)	− 1.039 (0.482)	0.064 (0.481)	1.698 (0.496)
*q11: Feeling of choking*				
Healthy	1.251 (0.132)	0.151 (0.812)	1.135 (0.806)	2.233 (0.865)
PLWHA	1.251 (0.132)	0.151 (0.812)	1.135 (0.806)	2.233 (0.865)
*q12: Hands trembling*				
Healthy	0.985 (0.084)	− 0.153 (0.572)	0.748 (0.571)	2.089 (0.646)
PLWHA	0.985 (0.084)	− 0.153 (0.572)	0.748 (0.571)	2.089 (0.646)
*q13: Shaky/unsteady*				
Healthy	1.360 (0.144)	− 0.163 (0.801)) (0.084)	0.913 (0.797)	2.119 (0.896)
PLWHA	1.360 (0.144)	− 0.163 (0.801)) (0.084)	0.913 (0.797)	2.119 (0.896)
*q14: Fear of losing control*				
Healthy	0.868 (0.072)	− 1.155 (0.536)	− 0.044 (0.525)	1.263 (0.592)
PLWHA	0.868 (0.072)	− 1.155 (0.536)	− 0.044 (0.525)	1.263 (0.592)
*q15: Difficulty in breathing*				
Healthy	1.161 (0.115)	0.227 (0.852)	1.429 (0.841)	2.436 (0.900)
PLWHA	1.161 (0.115)	0.227 (0.852)	1.429 (0.841)	2.436 (0.900)
*q16: Fear of dying*				
Healthy	0.539 (0.058)	− 0.698 (0.411)	− 0.068 (0.406)	0.838 (0.414)
PLWHA	0.539 (0.058)	− 0.698 (0.411)	− 0.068 (0.406)	0.838 (0.414)
*q17: Scared*				
Healthy	0.854 (0.071)	− 0.262 (0.514)	0.849 (0.508)	1.917 (0.537)
PLWHA	0.854 (0.071)	− 0.262 (0.514)	0.849 (0.508)	1.917 (0.537)
*q18: Indigestion*				
Healthy	0.800 (0.069)	0.421 (0.498)	1.339 (0.496)	2.359 (0.529)
PLWHA	0.800 (0.069)	0.421 (0.498)	1.339 (0.496)	2.359 (0.529)
*q19: Faint/lightheaded*				
Healthy	1.557 (1.262)	1.740 (1.280)	1.470 (1.212) 143.472	2.853 (2.245)
PLWHA	1.557 (1.262)	1.740 (1.280)	1.470 (1.212) 143.472	2.853 (2.245)
*q20: Face flushed*				
Healthy	0.626 (0.064)	1.290 (0.508)	2.208 (0.514)	2.986 (0.520)
PLWHA	0.626 (0.064)	1.290 (0.508)	2.208 (0.514)	2.986 (0.520)
*q21: Hot/ cold sweats*				
Healthy	0.785 (0.066)	0.577 (0.463)	1.329 (0.463)	2.573 (0.470)
PLWHA	0.785 (0.066)	0.577 (0.463)	1.329 (0.463)	2.573 (0.470)

b: factor loading, τ_i_: threshold parameters, SE: standard error

It should be noted that assessing DIF was also investigated for the two-factor BAI. The findings showed that the magnitudes of fit indices were exactly the same as one-factor model and the estimated parameters were very close to those represented in Table 4 (not reported here).

Table 5 represents the depression and anxiety scores (mean ± SD) of PLWHAs and healthy individuals. Although the anxiety scores did not differ significantly between these two groups, the depression scores of PLWHAs were significantly higher than those of healthy individuals.

**Table 5:** Comparison of depression and anxiety scores between PLWHAs and healthy individuals

Variable	PLWHA	Healthy	
	Mean ± SD	Mean ± SD	P value^¶^
Depression	10.45 ± 5.56	7.16 ± 4.95	< 0.001
Anxiety	8.34 ± 8.02	8.36 ± 8.12	0.974

^¶^P value based on independent samples t test

## Discussion

The current study investigated the measurement invariance of the CESD-10 and BAI across PLWHAs and healthy individuals, the issue which has never been investigated in the previous studies. Our findings provide evidence that the measurement invariance for valid comparison across healthy individuals and PLWHAs has been satisfied for the CESD-10 and BAI questionnaires. This implies that PLWHAs and healthy individuals perceived the meaning of the items in the CESD-10 and BAI questionnaires similarly. Furthermore, it can be concluded that the symptoms of depression and anxiety could not be misinterpreted as the symptoms of the disease or the side effects of medication.

Previous studies assessing the measurement invariance of several depression and anxiety questionnaires across healthy individuals and patients with different chronic conditions have reached conflicting results. In accordance with the results of the present study, some previous research has demonstrated the measurement invariance of the CESD, PHQ-9, PROMISD-8, K6 and GDS-15 across community sample and patients with neurologic, cognitive as well as arthritis problems [35, 49–51]. In contrast, Broekman et al. (2008) detected some items with DIF in GDS-15 questionnaire across healthy individuals and people with chronic illness [52]. In addition, in a study which examined the measurement invariance of the PHQ-9 across healthy individuals and patients with breast, lung, and colorectal cancers, two items with DIF were identified. Waller et al. (2005) also reported differential item functioning of Beck Depression Inventory questionnaire across women with breast cancers and women with major depression disorder [53]. Furthermore, it has been shown that Short Health Anxiety Inventory questionnaire was not an invariant measure across healthy individuals and patients with diabetes, breast cancer along with multiple sclerosis [36]. These contradictory results may be due to different questionnaires which have been applied in different studies as well as various kinds of health conditions studied in previous research.

It should be also noted that there is no consensus on factor structure of the BAI in previous studies. Although Beck (1988) originally reported a two-factor model for this instrument [43] thereafter, several studies have proposed different factor structures from one-factor to even five-factor model in different cultures [40, 54–56]. This issue may also lead to contrasting results in DIF assessment. In the present study, we considered the one-factor BAI based on the results of previous studies in Iran indicating that the one-factor model provided the best fit for the Persian version of the BAI [39, 40]. However, our findings supported both one- and two-factor models based on CCFA; more importantly, the results of DIF analysis was the same for both models.

Another important finding of the present study is that depression scores were significantly higher among PLWHAs as compared to healthy individuals. This result supports previous studies [1, 2, 57]. According to the proceeding research, stigma, poor family relations, limited psychological support, and lower socioeconomic status may explain the increased risk of depression in PLWHAs [1, 2, 21, 58–60].

Contrary to our expectations, this study showed that no significant difference was observed between anxiety scores of PLWHAs and healthy individuals. Although this result differs from some published studies which reported that anxiety was higher in PLWHAs as opposed to the general population [1–3, 6, 14, 57], it is consistent with other studies such as Prasithsirikul et al. (2017) and Sewell et al. (2000) [28, 60]. These contradictions in the results could be attributed to a variety of populations and instruments applied in different studies to ascertain the diagnosis of psychological disorders. One possible explanation for our findings might be that the mean duration of living with HIV in our sample was 5.52 ± 3.43 which is somehow a lengthy period in which PLWHAs have acquired skills to adapt to living with HIV so that they may no longer be afraid of dying from HIV [28, 60].

It is worth to mention some key advantages of using MG-MIMIC model in DIF assessment. First, as compared with MGCFA model, MG-MIMIC was able to control the potential confounding variables such as age, gender, and education which may affect the results of DIF analysis between healthy individuals and PLWHAs [44]. Second, unlike the MIMIC model, MG-MIMIC was able to detect non-uniform DIF in addition to uniform DIF. In addition, the purification of anchor items is another superiority of MG-MIMIC model in DIF detection process. The importance of this issue is due to the fact that ignoring purification results in over- or under-estimation in the number of DIF items [44, 45].

This study has some limitations that merit attention when interpreting the results. First, the factor loading of item 4 in the CESD-10 was lower than the suggested cut-point of 0.30. However, this result has not previously been described in a study examining the construct validity of the CESD-10 among PLWHAs in Canada [33]. Consequently, it would be beneficial to investigate the construct validity of the CESD-10 in the same sample in other languages and cultural contexts. Second, the sample of patients was gender-imbalance as the majority of PLWHAs was male. The result would be different if we matched PLWHAs and healthy individuals in terms of their gender. Moreover, the results of DIF may differ from one questionnaire to another; therefore, further investigations should be conducted to assess the measurement invariance of other depression and anxiety questionnaires. Different results would also be achieved if we assessed DIF among participants with other cultures, nationalities, or health conditions which can be an important issue for further research. Finally, Woods suggested that for reasonably powerful and accurate MIMIC results, sample size of focal (patients) group should be at least 100 with reference (healthy) group sample size of 500 [61]. Since no simulation study has been conducted regarding the sample size requirement in the MG-MIMIC model, we followed this outline for sample size determination in our study. It would be fruitful to investigate this important issue in future studies, too.

## Conclusions

In conclusion, the findings of this study suggest that the BAI and CESD-10 questionnaires can be considered as invariant measures across PLWHAs and healthy individuals, at least in our sample. More importantly, the measurement invariance of the questionnaires may ensure us that the higher depression scores of PLWHAs in comparison to healthy individuals is a real difference. It is highly recommended that health professionals develop therapeutic interventions and psychological supports to promote the mental health of PLWHAs that alleviate their depression symptoms.

## Data Availability

The datasets used and analyzed during the current study is available from the corresponding author on reasonable request (pjbiostat@gmail.com).

## Abbreviations

BAI: Beck Anxiety Inventory
CESD-10: 10-Item Centre for Epidemiological Studies Depression Scale
PLWHA: People living with HIV/AIDS
MG-MIMIC: Multi-group multiple-indicator multiple-causes
VCT: Voluntary counselling and testing centre
DIF: Differential item functioning
RMSEA: Root mean square error of approximation
TLI: Tucker–Lewis index
CFI: Comparative fit index
WLSMV: Mean and variance-adjusted weighted least square
